# Organic Dye-Derived N, S Co-Doped Porous Carbon Hosts for Effective Lithium Polysulfide Confinement in Lithium–Sulfur Batteries

**DOI:** 10.3390/nano11112954

**Published:** 2021-11-04

**Authors:** Eunji Kim, Albert S. Lee, Taewoong Lee, Hyeok Jun Seo, Seongwook Chae, Kihyun Kim, Jun-Woo Park, Seung Geol Lee, Jin Hong Lee

**Affiliations:** 1 School of Chemical Engineering, Pusan National University, Busan 46421, Korea; eunjikim@pusan.ac.kr (E.K.); wmfrlwk12@pusan.ac.kr (T.L.); seohj@pusan.ac.kr (H.J.S.); swchae0708@pusan.ac.kr (S.C.); 2 Materials Architecturing Research Center, Korea Institute of Science and Technology, Seoul 02792, Korea; aslee@kist.re.kr; 3 Department of Materials Engineering and Convergence Technology, Gyeongsang National University, Jinju 52828, Korea; 4 Next Generation Battery Research Center, Korea Electrotechnology Research Institute (KERI), Changwon 51543, Korea

**Keywords:** lithium sulfur batteries, organic dye, graphene, heteroatom doping

## Abstract

Lithium–sulfur batteries are considered as attractive candidates for next-generation energy storage systems originating from their high theoretical capacity and energy density. However, the severe shuttling of behavior caused by the dissolution of lithium polysulfide intermediates during cycling remains a challenge for practical applications. Herein, porous carbon materials co-doped with nitrogen and sulfur atoms were prepared through a facile hydrothermal reaction of graphene oxide and methylene blue to obtain a suitable host structure for regulating the lithium polysulfide shuttling behavior. Experimental results demonstrated that the abundant heteroatom-containing moieties in the carbon frameworks not only generated favorable active sites for capturing lithium polysulfide but also enhanced redox reaction kinetics of lithium polysulfide intermediates. Consequently, the corresponding sulfur composite electrodes exhibited excellent rate performance and cycling stability along with high Columbic efficiency. This work highlights the approach for the preparation of nitrogen and sulfur co-doped carbon materials derived from organic dye compounds for high performance energy storage systems.

## 1. Introduction

With the noticeably increasing demands for energy storage systems and electric vehicles, lithium–sulfur (Li-S) batteries have been widely investigated as promising next-generation battery systems due to their overwhelming electrochemical performances, such as high theoretical specific capacity (1675 mAh/g) and energy density (2800 Wh/L) [1,2]. Furthermore, sulfur has the additional advantages of natural abundance and cost-effective as well as environmentally friendly resources [3]. Despite these advantages over the conventional lithium-ion batteries, the practical application of the rechargeable Li-S batteries is still hindered by many problems, in particular the limited utilization of sulfur active materials and poor cycling performance [4]. During the repeated charge–discharge processes, the lithium polysulfide species (Li_2_Sx, 4 ≤ x ≤ 8) formed from the cathode are progressively solvated in the organic electrolyte and diffuse between the anode and the cathode, resulting in parasitic reactions [5]. This phenomenon is referred to as a shuttling behavior, which causes loss of an active material and fast capacity fading. In addition, Li-S batteries suffer from the inherent poor electrical conductivity of sulfur and the discharge products of Li_2_S/Li_2_S_2_, leading to sluggish reaction kinetics [6]. In addition, the large volume expansion of ~80% upon full lithiation leads to the formation of an unstable electrode/electrolyte interface layer [7].

To solve these issues of the Li-S batteries, various strategies of incorporating sulfur species in a carbonaceous host material derived from micro/mesoporous carbons [8], carbon nanotubes (CNTs) [9], graphene [10], and carbon nanofiber [11] have been investigated. The physical/chemical confinement of sulfur in these host materials not only mitigates the shuttling behavior by preventing the dissolution of lithium polysulfide but also promotes redox reaction kinetics with the help of conductive pathways provided by the carbon framework. Moreover, the porous carbonaceous materials with large specific surface area accommodate a large amount of sulfur and offer a lot of active sites for electrochemical reactions, which leads to improved lithium storage behavior [12,13]. However, non-polar carbon materials exhibit only weak physical adsorption to the polar lithium polysulfides, resulting in an unsatisfactory suppression of lithium polysulfide shuttling behavior during long-term cycling [14].

Therefore, it is essential to introduce a reasonable design of carbon-based materials with polar moieties to provide strong chemical interactions with the lithium polysulfide. To date, tremendous efforts have been devoted to develop advanced carbon composite materials based on many different types of polymers [15], metal oxides [16,17], and metal-organic frameworks [18]. While these approaches have been demonstrated to be effective for the chemisorption of lithium polysulfides, the limited conductivity of these materials is still not sufficient to achieve good rate capability. As such, modifications of the carbon materials with heteroatoms, such as boron, oxygen, sulfur, nitrogen, and phosphorus, have been explored [19,20,21]. Of these heteroatoms, nitrogen and sulfur atoms have proved to effectively increase the adsorption capability of carbon materials via strong chemical interactions, with the π-conjugated structures ensuring an efficient electron transport to promote redox reaction kinetics [22].

In this study, microporous nitrogen and sulfur co-doped reduced graphene oxides (rGO) regulating the lithium polysulfide shuttling behavior were prepared as a host material for Li-S cathode through facile hydrothermal and freeze-drying treatments. A cationic dye, methylene blue (MB) was chosen as a precursor for heteroatom doping, because the heterocyclic aromatic structure with positively charged nitrogen or sulfur moiety of the MB could allow for favorable interactions with GO aqueous suspensions, giving rise to well-developed porous graphene structures with ample nitrogen- and sulfur-containing moieties. The sulfur cathodes were able to deliver higher specific capacity and better cycling performance during cycling when compared with those of rGO cathodes. Spectroscopic analysis and electrochemical performance evaluation coupled with redox reaction kinetics investigations revealed that the heteroatom-containing moieties in the carbon frameworks were able to provide facilitated charge transport and alleviate lithium polysulfide shuttling behavior, leading to the enhanced Li-S battery performances.

## 2. Materials and Methods

### 2.1. Preparation of Nitrogen and Sulfur Co-Doped Carbon via Hydrothermal Method

To prepare the nitrogen and sulfur co-doped carbon materials, different amounts of methylene blue (MB, Sigma-Aldrich, St. Louis, MO, USA) were dissolved in graphene oxide (GO) aqueous dispersion (5 mg/mL) and vigorously sonicated for 30 min. MBGOx represent the precursor mass ratio of MB:GO = x:100. The mixed solution was transferred into a Teflon-lined autoclave and heated 180 °C for 24 h, followed by cooling to room temperature. The hydrogel mixture was repeatedly washed with deionized water and freeze-dried under a vacuum. For comparison, the rGO sample was also prepared via the same process without the addition of MB.

### 2.2. Material Characterizations

Zeta potential (Zetasizer, Malvern Panalytical, Malvern, UK) measurement was performed to identify surface charges. Scanning electron microscope (SEM, SUPRA25, Zeiss, Oberkochen, Germany) was used to analyze the morphology of the MBGOs and rGO materials. The elemental distribution of MBGO20 were observed using a transmission electron microscope (TEM, TALOS F200X, FEI, Hillsboro, OR, USA) equipped with an energy dispersive X-ray spectroscopy (EDS). X-ray diffraction (XRD, Xpert 3, Malvern Panalytical, Malvern, UK) and Raman spectroscopy (RAMANtouch, Nanophoton, Minato, Japan) were performed to carbon crystallographic properties. The X-ray photoelectron spectroscopy (XPS, AXIS SUPRA, Kratos Analytical Ltd., Manchester, UK) was carried out to the surface chemical binding state.

### 2.3. Electrochemical Measurements

The sulfur/MBGO composites were prepared by heating a mixture of sulfur and MBGO20 with a mass ratio of 3:1 at 155 °C for 12 h in a stainless-steel vessel (Figure S1). The sulfur cathode was prepared by stirring the slurry mixture of 70 wt% of sulfur/MBGO20 composites, 10 wt% of the polyvinylidene fluoride (PVDF) binder and 10 wt% of Super-P in N-methyl-2-pyrrolidone (NMP) solvent. The slurry was coated on an aluminum current collector, and then dried at 80 °C for 24 h under a vacuum. Similarly, a cathode with the rGO sample was prepared using the same procedure. The loading of sulfur mass was about 1.0 mg/cm^2^. The as-prepared cathode, lithium metal anode and polypropylene separator were assembled into a CR2032 coin cell with an electrolyte of 1.0 M lithium bis(trifluoromethane)sulfonamide lithium (LiTFSI) and 0.1 M lithium nitrite (LiNO_3_) additive in a mixture of 1,3-dioxolane (DOL) and 1,2-dimethoxyethane (DME) (1:1 by volume). The electrochemical performances were conducted using an automatic charge–discharge instrument (WBCS3000, WonATech Co., Seoul, Korea) in a voltage window of 1.8–2.8 V. Cyclic voltammetry (CV) and electrochemical impedance spectroscopy (EIS) measurements were performed with Biologic BCS-805 and Biologic SP-150 workstation, respectively. EIS tests were carried on the frequency ranges from 1.00 MHz to 0.01 Hz with a disturbance amplitude of 10 mV.

## 3. Results and Discussion

**Figure 1:**
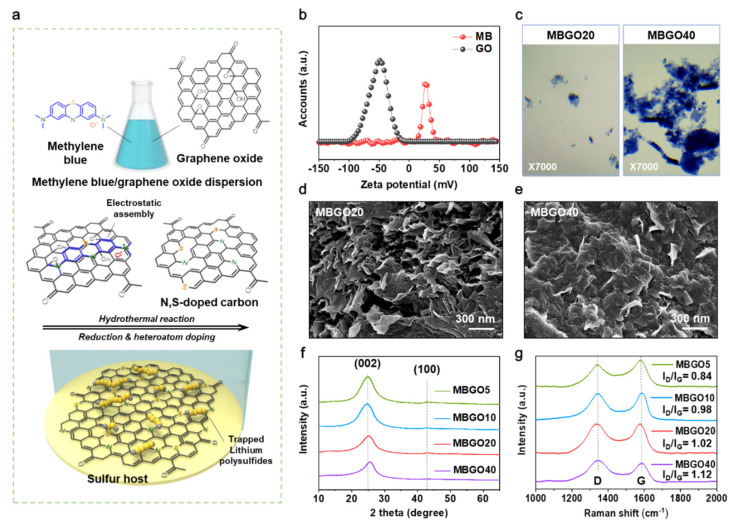
(**a**) Schematic of the preparation process of N, S–doped carbon as a sulfur host. (**b**) Zeta potential of graphene oxide and methylene blue. (**c**) Optical microscopy observations of the bottom of vial for MBGO20 and MBGO40 solutions. SEM image of (**d**) MBGO20 and (**e**) MBGO40. (**f**) XRD patterns and (**g**) Raman spectra of MBGOs with different MB contents.

As schematically illustrated in Figure 1a, porous N, S co-doped rGO host materials regulating the lithium polysulfide shuttling behavior were prepared via a simple hydrothermal method using methylene blue (MB) as a precursor for doping of heteroatoms onto the graphene layers. The MB is a heterocyclic aromatic organic dye that contains a delocalized charge distribution in aqueous solution; only weak positive charge characteristic was detected from the zeta potential measurement. (Figure 1b). We speculated that negatively charged oxygen-containing functional groups on the surface of GO sheets could induce a complexation of GO and MB through electrostatic interactions, with π-π bond providing additional interactions for the interfacial assembly. To investigate the interaction of MB and GO, different amounts of MB aqueous solutions were added to a GO suspension and left for several hours. We found that these mixtures spontaneously assembled with increasing the content of MB. For the MBGO20, small aggregates were observed as presented by optical microscopy. (Figure 1c) Remarkably, after increasing the content of methylene blue of up to 40%, MBGO40, relatively larger and more aggregates were obviously visible at the bottom of the vial, which could be a consequence of complete covering of the charge of the GO sheets, leading to the formation of highly aggregated precipitates out of the solutions. Thus, we considered that the addition of excessive amount of MB would be a negative influence on achieving a homogeneity and porous structure of host materials during the hydrothermal reaction.

The morphologies of the MBGO were investigated by scanning electron microscopy (SEM) with the images displayed in Figure 1d,e. After thermal expansion by the hydrothermal reaction, the rGO nanosheet formed a three-dimensional network with slight folds and crumpled edges (Figure S2), while maintaining the well-defined porous structure. Similarly, the MBGO samples revealed the layered and porous network structures, but the nanosheets were progressively stacked to form a compact structure with dense and continuous surface morphologies upon increasing the content of MB.

The X-ray diffraction (XRD) analysis was performed to better understand the microstructure of the MBGOs. As shown in Figure 1f, the MBGOs exhibited two broad peaks around 24° and 43°, corresponding to the (002) and (100) reflections of planes in the disordered carbon materials, indicative of the partially restacked rGO [23]. We observed that the (002) peak position shifted toward the higher angle of 2θ with increasing the content of methylene blue compared to the pristine rGO (Figure S3), suggesting that the introduction of methylene blue decreases the interlayer distance of the MBGOs [24]; this is generally coupled with the deoxidization of oxygen functional group due to the heteroatom (e.g., B, N, and S) doping in the graphitic layer. The structural disorder tendency of MBGOs was also investigated by Raman spectroscopy. As shown in Figure 1g, the MBGOs exhibited two distinctive peaks at 1320 cm^−1^ and 1590 cm^−1^ related to the D (defects and disorder in the graphitic layer) and G band (sp^2^ hybridized carbon atoms) respectively, indicating a presence of disordered graphitic layers [25]. The corresponding intensity ratio of the D and G bands (*I*_D_/*I*_G_) gradually increased with the rise of methylene blue content, which could be ascribed to the incorporation of heteroatoms into the carbon framework [24].

**Figure 2:**
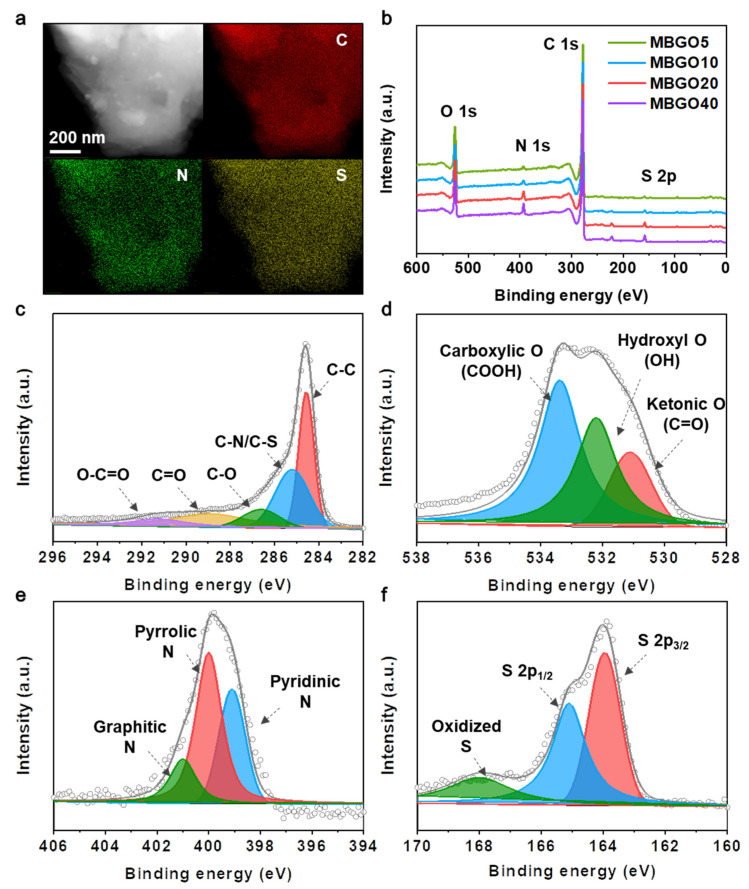
(**a**) STEM image and corresponding elemental mapping images of C, N and S for MBGO20. (**b**) XPS survey spectra with different MB contents. The high-resolution XPS spectra of (**c**) C 1s, (**d**) O 1s, (**e**) N 1s and (**f**) S 2p of the MBGO20.

Figure 2a displays the STEM images and corresponding energy-dispersive X-ray spectroscopy (EDS) mapping images of the MBGO20. Uniform distribution of carbon, nitrogen, and sulfur elements over the entire carbon structure was observed, again demonstrating that the nitrogen and sulfur atoms were successfully incorporated in the carbon matrix through the hydrothermal treatment.

The XPS analysis was performed further to investigate the surface element composition and chemical state of MBGOs depending on the contents of methylene blue. As shown in Figure 2b, the XPS survey spectrum exhibited the presence of carbon, nitrogen, sulfur, and oxygen species. The amount of nitrogen and sulfur atoms increased continuously with the increasing the amount of methylene blue, accompanied by decreasing the content of oxygen element as listed in Table S1. The high-resolution C 1s, O 1s, N 1s, and S 2p spectrum of MBGO20 are displayed in Figure 2c–f, respectively. The C 1s spectrum (Figure 2c) exhibited the characteristic peaks located at 284.5 and 285.2 eV, which could be indexed to the C–C groups constituting the carbon structure and the C–N/C–S groups formed by heteroatoms doping, respectively. In addition, small peaks for C–O, C=O, and O–C=O originated from oxygen functional groups were observed at 286.6 eV, 289.0 eV, and 291.3 eV [26]. The O 1s spectrum could be deconvoluted into C=O, –OH, and COOH three peaks centered at 531.1, 533.4, and 533.4 eV, respectively (Figure 2d) [27]. The binding state of nitrogen and sulfur containing moieties was also investigated. As shown in Figure 2e, the N 1s spectrum was fitted by three typical peaks, including pyridinic N (N–6, 399.1 eV), pyrrolic N (N–5, 400.1 eV), and graphitic N (N–Q, 401.0 eV) [28]. The S 2p spectrum exhibited three sub-peaks located at 163.9, 165.2, and 167.9 eV, corresponding to the S 2p_3/2_, S 2p_1/2_, and oxidized S, respectively [29]. Previous studies have proved that the incorporation of nitrogen and sulfur containing moieties into the carbon materials could not only offer favorable physical and/or chemical trapping sites to alleviate the lithium polysulfide shuttling behavior, but also expedite the redox reaction kinetics of sulfur species, resulting in the high electrochemical performances [22,30]. Although the heteroatom content of the MBGO40 was estimated to be slightly lower than that of the MBGO20, we considered that the highly stacked MBGO40 hybrid structure that would hamper ion transport during cycling and decrease the contact sites between the heteroatoms and the lithium polysulfide when employed as sulfur hosts for Li–S batteries.

**Figure 3:**
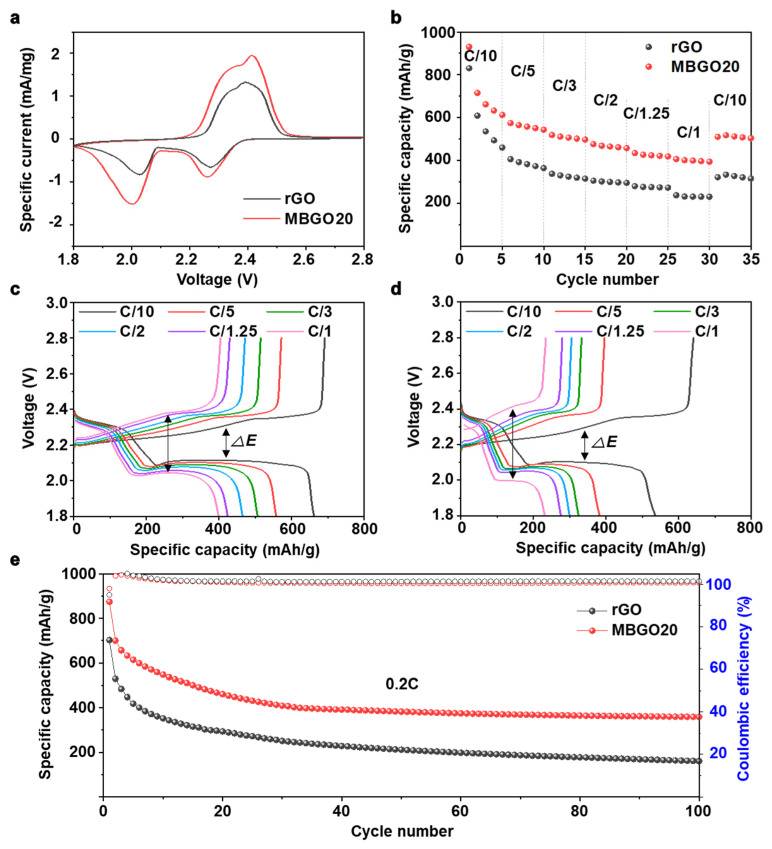
(**a**) CV curve of MBGO20 and rGO cells at 0.1 mV/s in the voltage window of 1.8 to 2.8 V. (**b**) Rate capability and galvanostatic charge–discharge profiles of (**c**) MBGO20 and (**d**) rGO cells at various current rates. (**e**) Cycling performance and Coulombic efficiency at 0.2 C.

As such, the electrochemical performance was evaluated for the Li–S cells fabricated with MBGO20 cathode. The cyclic voltammetry (CV) was initially carried out to identify redox reaction behavior of the MBGO20 cathode at a current rate of 0.1 mV/s. For comparison, the CV curve for the cell with pristine rGO cathode is also provided. As shown in Figure 3a, during the cathodic scan, two peaks were observed at around 2.1 and 2.3 V, indicating the reduction of sulfur (S_8_) to long-chain lithium polysulfide (Li_2_S_x_) and the reduction of long-chain lithium polysulfide (Li_2_S_X_) to insoluble lithium polysulfide (Li_2_S_2_/Li_2_S), respectively. In the anodic scan, the oxidation peaks at around 2.4 V and 2.5 V correspond to the conversion of Li_2_S_2_/Li_2_S into long-chain Li_2_S_X_ and eventually to S_8_ [31]. Obviously, the cell with MBGO20 cathodes showed significantly higher and sharper redox peaks along with larger area than those of the rGO cathode, suggesting that the MBGO20 cathode was able to improve redox reaction kinetics and capacitive behavior.

The rate capability of the cells with MBGO20 and rGO cathode was measured at different current rates from C/10 to 1 C. As shown in Figure 3b, MBGO20 cathode showed higher specific capacities at all current rates when compared to the rGO cathode, and the difference in specific capacity was noticeable with increasing current rates. Furthermore, when returned to the current rate of C/10, the specific capacity of MBGO20 cathode recovered reasonable specific capacities, indicating high electrochemical reversibility. The galvanostatic charge–discharge profiles of MBGO20 and rGO cathode at various current rates are compared, where the two well-defined charge and discharge plateaus were observed at a mild condition of 1/10 C, which was in good agreement with the CV results. As presented in Figure 3c,d, we observed that there is a difference between the charge capacity and the discharge capacity, especially in the first cycle. This result indicates the low Coulombic efficiency during the initial cycling, which could be attributed to the formation of a solid electrolyte interphase (SEI) layer. [5] Additionally, we were not able to suppress completely the parasitic reactions derived from the diffusion of lithium polysulfide dissolved in electrolytes [32]. Nonetheless, the MBGO20 cathode showed much improved electrochemical performances and Coulombic efficiency compared to the rGO cathode possibly due to well-developed porous graphene structures with ample nitrogen- and sulfur-containing moieties, which suggests that the MBGO20 cathode was able to mitigate the parasitic reactions to the electrode. In addition, the voltage differences between the charge–discharge curves of the rGO cathode were found to be larger than those of the MBGO20 cathode, and the differences were more prominent with increasing current rates, with the discharge plateaus gradually disappearing. By comparison, the MBGO20 cathode showed a relatively stable charge–discharge behavior with lower polarization, which further indicates the enhanced reaction kinetics.

Figure 3e displays the cycling performance and CE of the cells with MBGO20 and rGO cathodes at a current rate of 0.2C. As expected, the MBGO20 cathode was able to deliver improved cycling performance compared to the rGO cathode. The rGO cathode exhibited a limited capacity retention of about 21%, while the MB20 cathode showed a double capacity retention. In addition, CE of the MBGO20 cathodes was observed to be close 99% during cycles, confirming excellent cycling stability. We considered that the introduction of heteroatom into the carbon framework not only gives rise to fast redox reaction kinetics but also mitigates the shuttling behavior of lithium polysulfide derived from a polar interaction during cycling, which in turn improves utilization of active materials and thus provide the higher electrochemical performances.

**Figure 4:**
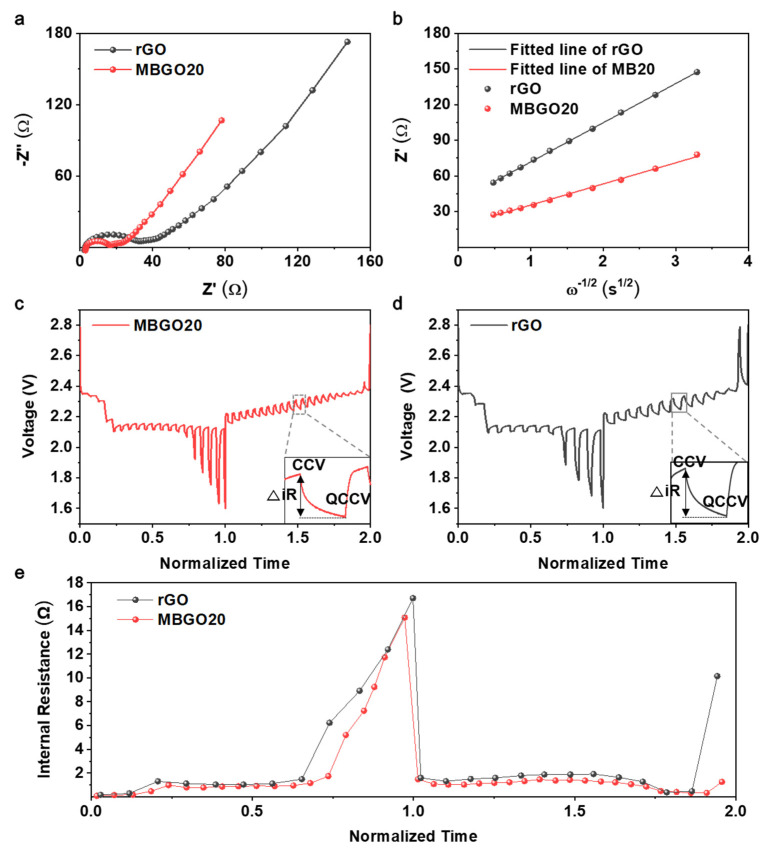
(**a**) Nyquist plots of MBGO20 and rGO cathodes. (**b**) Relationship between Z’ and ω^−1/2^ in the low-frequency region. GITT curves of (**c**) MBGO20 and (**d**) rGO cathode at 0.02 C. (**e**) Internal resistance based on GITT.

To better understand the redox reaction kinetics, electrochemical impedance spectra (EIS) measurements were conducted after cycling, and the relevant equivalent circuit model is shown in Figure S4. As displayed in Figure 4a, both Nyquist plots exhibited two semicircles at high- to medium-frequency ranges, an oblique line at low frequency. The first semicircle is a combination of bulk resistance of the electrolyte (*R*_0_) and insulating surface layer (*R*_surf_), and the second semicircle relates to charge-transfer resistance (*R*_ct_). In addition, the oblique line corresponds to the Warburg impedance (*W*_s_) associated with the diffusion of lithium ions in the electrode [33]. It can be noticed that the MBGO20 cathodes showed smaller *R*_surf_ and *R*_ct_ values, which demonstrates the suppressed formation of insulating Li_2_S_2_/Li_2_S layer and excellent lithium polysulfide conversion kinetics. Additionally, we speculated that the lone pair electrons in nitrogen and sulfur would form conjugation structures which improve the electrical conductivity of the MBGO20, leading to the reduced charge transfer resistance and fast redox reaction kinetics, as reported previously [34].

The diffusion coefficient of lithium ions (*D*_Li_^+^) in the low-frequency region was calculated further by the following Equation (1) [35,36]:

*D*_Li_^+^ = *R*^2^*T*^2^/2*A*^2^*n*^4^*F*^4^*C*^2^*σ*^2^ (1)

where *R*, *T*, *A*, *n*, *F,* and *C* are gas constant, absolute temperature, cathode surface area, electrons number per molecule in redox reaction, Faraday constant and lithium ion concentration in electrolyte, respectively. σ is Warburg coefficient, calculated according to the following Equation (2):

*Z*’ = *R*_0_ + *R*_ct_ + *σω*^−1/2^ (2)

where *ω* represents the angular frequency. The linear relationship between *Z*’ and *ω*^−1/2^ is shown in Figure 4b, and the Warburg coefficient of the MBGO20 cathode found to be smaller than that of the rGO cathode, demonstrating the improved redox reaction kinetics and Li^+^ diffusion for the MBGO20 cathode. The *D*_Li_^+^ values of MBGO20 and rGO cathodes were calculated to be 5.5 × 10^−12^ cm^2^/s and 1.6 × 10^−12^ cm^2^/s, respectively.

Subsequently, the galvanostatic intermittent titration technique (GITT) was performed at 0.02 C in order to further investigate the effect of MBGO on the lithium polysulfide conversion kinetics. As presented in voltage fluctuations (Figure 4c,d), during charge and discharge processes, the MBGO20 cathode exhibited lower cell polarizations that are clearly evident in the magnified pulse compared with the rGO cathode, which is in accordance with the charge transfer behavior. For stricter comparison, the internal resistances of MBGO20 and rGO cathodes during cell operation were calculated using the following Equation (3) [37,38]:

∆*R*_internal_(Ω) = |∆*V*_QOCV-CCV_|/*I*_applied_ (3)

where ∆*V* represents the voltage difference between the closed-circuit voltages (OCV) and the quasi-open-circuit voltage (QOCV), and *I*_applied_ represents the applied current. ∆*R*_internal_ is defined as the internal resistance of cell related to lithium polysulfide conversion during charge and discharge. As shown in Figure 4e, the internal resistance values for the MBGO20 cathode observed to be the lower during both charge and discharge processes compared with rGO cathode, which demonstrates a significant improvement in the redox kinetics for lithium polysulfide through rapid electron and lithium-ion transport.

**Figure 5:**
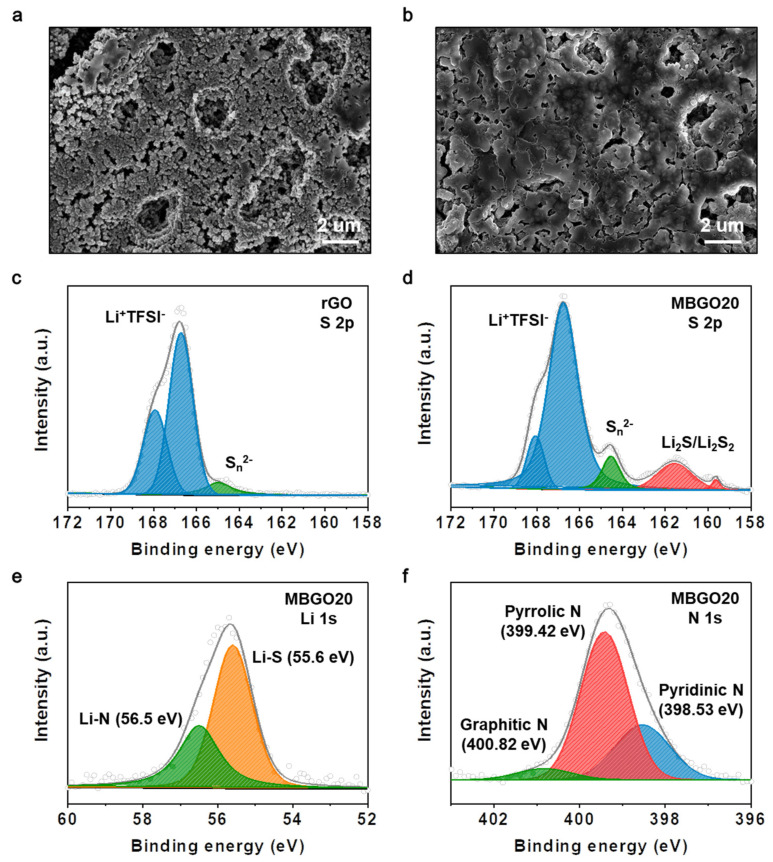
SEM images of the lithium metal anode surface of (**a**) rGO and (**b**) MBGO20 cathode after 100 cycles. High–resolution XPS spectra of (**c**) S 2p of rGO cathode and (**d**) S 2p, (**e**) Li 1s and (**f**) N 1s of MBGO20 cathode after cycling.

After 100 cycles, the surface morphology of the lithium metal anode was observed to monitor the change in lithium polysulfide shuttling behavior using scanning electron microscopy (SEM). As presented in Figure 5a, the lithium metal anode surface of the rGO cathode showed a lot of irregular dendritic particles and holes along with rough passivation layers, which resulted from the continuous lithium polysulfide shuttling behavior and decomposition of the electrolyte. In comparison, the lithium metal anode of MBGO20 cathode was observed to be smoother and flatter surface as displayed in Figure 5b. These results suggest that the MBGO20 cathode was able to effectively alleviate the dendrite growth on the surface of lithium anode and lithium polysulfide shuttling behavior.

We performed the lithium polysulfide confinement experiment under ambient conditions. For the test, a Li_2_S_6_ solution of red-brown color was prepared by mixing a 1:5 molar ratio of Li_2_S and sulfur in DOL/DME solution (1:1, *v*/*v*). After adding the MBGO20 powder into the Li_2_S_6_ solution, the solution showed color fading. This result indicates that the MBGO20 was able to trap the lithium polysulfide through strong chemical and physical interactions. In order to further investigate the suppressed lithium polysulfide shuttling behavior for the MBGO20 cathode, XPS analysis was performed after 100 cycles. As shown in Figure 5c,d, the peaks related to Li^+^TFSI^−^ electrolyte salt at 170–166 eV detected for both two cathodes in the S 2p spectra. However, the MBGO20 cathode clearly exhibited a series of distinct peaks corresponding to polysulfide (S_n_^2−^, 165–164 eV) and Li_2_S/Li_2_S_2_ (164–160 eV), which indicated that a high degree of lithium polysulfide was anchored in the MBGO20 cathode [39]. We considered that the differences in lithium polysulfide shuttling behavior were closely related to the introduction of heteroatoms in the carbon materials. Possibly the electronegative nitrogen and sulfur containing moieties in the MBGO20 were able to offer efficient chemical interactions for the lithium polysulfide, with a lot of structural defects generated after heteroatoms doping providing an increase in physical adsorption sites for the lithium polysulfide. The XPS spectra of Li 1s and N 1s of MBGO20 cathode further demonstrated the alleviated lithium polysulfide shuttling behavior. As shown the Figure 5e, the Li 1s spectrum was deconvoluted into two peaks at 55.6 eV and 56.5 eV, which relates to the Li–S bond derived from lithium polysulfide and the Li–N bond from the interaction between the lithium polysulfide and nitrogen atom moieties [40]. In addition, the N 1s spectrum was divided into three peaks of pyridinic N (398.53 eV), pyrrolic N (399.42 eV), and graphitic N (400.82 eV) as presented in Figure 5f. Compared with the N 1s XPS spectra of Figure 2e, we observed a peak shift towards higher binding energy values for pyridinic N (+0.57 eV), pyrrolic N (+0.58), and graphitic N (+0.18 eV), due to the favorable polar interactions [41,42]. Therefore, the MBGO20 with active nitrogen and sulfur containing moieties was able to achieve the mitigated lithium polysulfide behavior, leading to the improved reactivation and reutilization of active materials, which in turn contributes to the enhanced electrochemical performances.

## 4. Conclusions

In summary, we rationally prepared a series of heteroatom-doped porous rGO to utilize as host materials for the sulfur cathode. A simple hydrothermal process of the methylene blue and graphene oxide simultaneously enabled the reduction of graphene oxide and the incorporation of N and S atoms into the carbon framework. The electronegative N, S–containing moieties of MBGOs were able to alleviate the polysulfide shuttling behavior through the combining effect of physical and chemical adsorptions, with promoting the redox reaction kinetics of lithium polysulfide intermediates. Thus, electrochemical investigation revealed that the sulfur cathode based on MBGO20 displayed the improved rate capability and cycling stability along with high along with high Columbic efficiency when compared to those with the rGO based cathode. This work provides an effective and facile method for the preparation of organic dye-derived carbon host materials for high performance Li–S batteries.

## Data Availability

The data presented in this study are available on request from the corresponding author.

## Supplementary Materials

The following are available online at https://www.mdpi.com/article/10.3390/nano11112954/s1, Figure S1: TGA thermograms of carbon composite and elemental sulfur, Figure S2: SEM images of (a) rGO, (b) MBGO5 and (c) MBGO10, Figure S3: XRD pattern of rGO sample, Figure S4: Equivalent circuit model of after cycling, Figure S5: Digital photo image of the confined lithium polysulfides within MBGO20, Table S1: Elemental ratios of MBGOs with different methylene blue contents.
