# Supplementary material for: Organic Dye-Derived N, S Co-Doped Porous Carbon Hosts for Effective Lithium Polysulfide Confinement in Lithium–Sulfur Batteries
Source: Nanomaterials (Basel). 2021 Nov 4;11(11):2954. doi: 10.3390/nano11112954 (PMC8624343; doi:10.3390/nano11112954)
Supplement: Supplementary file 1 [file nanomaterials-11-02954-s001.zip › nanomaterials-1443153-Supplementary.pdf]

Supplementary Material

Organic Dye-Derived N, S Co-doped Porous Carbon Hosts for Effective Lithium Polysulfide Confinement in Lithium–Sulfur Batteries

Eunji Kim [1], Albert S. Lee [2], Taewoong Lee [1], Hyeok Jun Seo [1], Seongwook Chae [1], Kihyun Kim [3,*], Jun-Woo Park [4,*], Seung Geol Lee [1,*] and Jin Hong Lee [1,*]

[1] School of Chemical Engineering, Pusan National University, Busan 46421, Korea; eunjikim@pusan.ac.kr (E.K.); wmfrlwk12@pusan.ac.kr (T.L.); seohj@pusan.ac.kr (H.J.S.); swchae0708@pusan.ac.kr (S.C.)

[2] Materials Architecturing Research Center, Korea Institute of Science and Technology, Seoul 02792, Korea; aslee@kist.re.kr

[3] Department of Materials Engineering and Convergence Technology, Gyeongsang National University, Jinju 52828, Korea

[4] Next Generation Battery Research Center, Korea Electrotechnology Research Institute (KERI), Changwon 51543, Korea

* Correspondence: kihyun@gnu.ac.kr (K.K.); parkjw@keri.re.kr (J.-W.P.); seunggeol.lee@pusan.ac.kr (S.G.L.); jinhong.lee@pusan.ac.kr (J.H.L.)

Citation: Kim, E.; Lee, A.S.; Lee, T.; Seo, H.J.; Chae, S.; Kim, K.; Park, J.-W.; Lee, S.G.; Lee, J.H. Organic Dye-Derived N, S Co-Doped Porous Carbon Hosts for Effective Lithium Polysulfide Confinement in Lithium–Sulfur Batteries. *Nanomaterials* **2021**, *11*, 2954. https://doi.org/10.3390/10.3390/nano11112954

Academic Editor(s): Marc Cretin, Sophie Tingry, Zhenghua Tang

Received: 15 October 2021
Accepted: 02 November 2021
Published: 04 November 2021

Publisher's Note: MDPI stays neutral with regard to jurisdictional claims in published maps and institutional affiliations.

Copyright: © 2021 by the authors. Submitted for possible open access publication under the terms and conditions of the Creative Commons Attribution (CC BY) license (https://creativecommons.org/licenses/by/4.0/).

Sample	C 1s (atomic %)	O 1s (atomic %)	N 1s (atomic %)	S 2p (atomic %)
MBGO5	71.07	26.98	1.36	0.59
MBGO10	71.58	25.27	1.91	0.97
MBGO20	72.18	21.10	4.39	2.33
MBGO40	72.30	18.56	5.82	3.32

Table S1. Elemental ratios of MBGOs with different methylene blue contents.

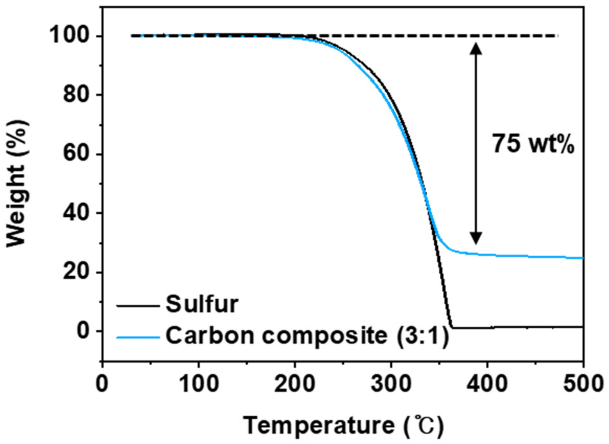

Figure S1. TGA thermograms of carbon composite and elemental sulfur.

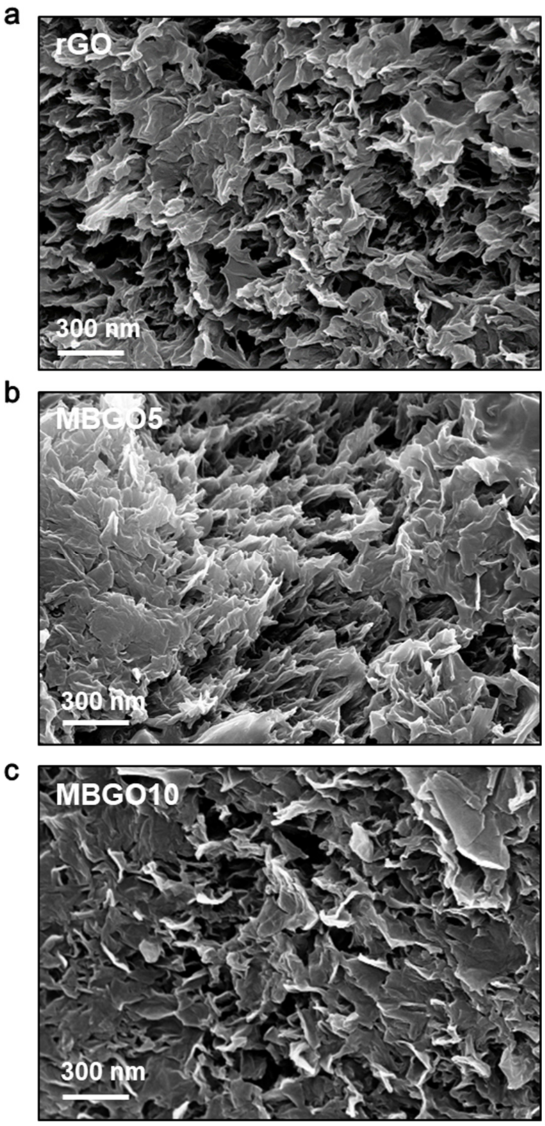

Figure S2. SEM images of (**a**) rGO, (**b**) MBGO5 and (**c**) MBGO10.

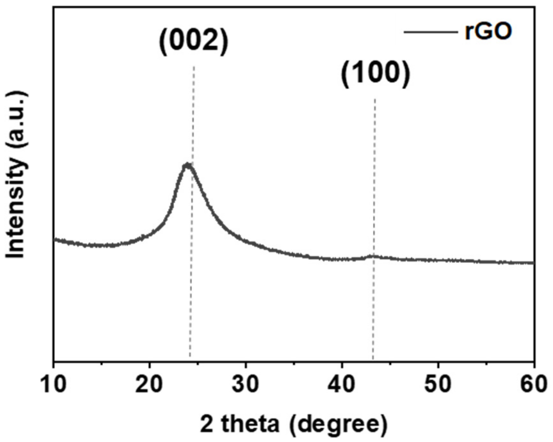

Figure S3. XRD pattern of rGO sample.

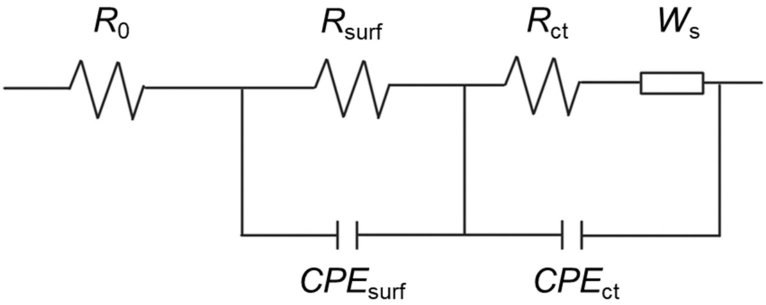

Figure S4. Equivalent circuit model of after cycling.

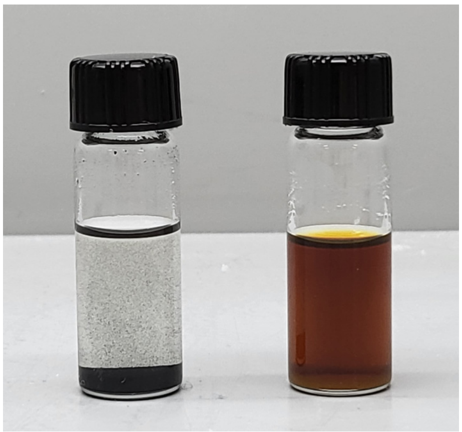

Figure S5. Digital photo image of the confined lithium polysulfides within MBGO20.
